# Age-related morphometrical peculiarities of Lithuanian women’s primordial ovarian follicles

**DOI:** 10.1186/s12958-018-0384-4

**Published:** 2018-07-14

**Authors:** Kristina Lasiene, Donatas Gasiliunas, Nomeda Juodziukyniene, Aleksandras Vitkus

**Affiliations:** 1 Department of Histology and Embryology, Medical Academy, Lithuanian University of Health Sciences, A. Mickeviciaus str. 9, LT-44307 Kaunas, Lithuania; 2 Kaunas Division of State Forensic Medicine Service, Perlojos str. 28, LT-45305 Kaunas, Lithuania; 3 Department of Veterinary Pathobiology, Veterinary Academy, Lithuanian University of Health Sciences, Tilzes str. 18, LT-47181 Kaunas, Lithuania

**Keywords:** Morphometrical, Primary oocyte, Primordial ovarian follicle, Women

## Abstract

**Background:**

For the first time, thorough morphometrical measurements of primordial ovarian follicles were performed and their age-related changes were investigated in Lithuanian women of the reproductive age.

**Methods:**

Ovaries of dead women (*n* = 30) were divided into six age groups: 15–20 years old, 21–25 years old, 26–30 years old, 31–35 years old, 36–40 years old and 41–46 years old. Histological slides of left and right ovaries were stained using haematoxylin-eosin and periodic acid–Schiff (PAS) staining methods. The morphometrical measurements of 10 primordial ovarian follicles of the left and right ovary of each woman were made from microphotographs.

**Results:**

The diameter of primordial ovarian follicles increased in groups of women from 15 years old to 35 years old and decreased in the groups from 36 years old to 46 years old. The area of primordial ovarian follicles increased in the groups of women until 35 years old. It decreased in the groups of women older than 36 years. The follicular basement membrane thickened from 1.29 ± 0.11 μm to 1.43 ± 0.18 μm with increasing age of women. The diameter of primary oocytes enlarged until 35 years and then began to decrease. The area of primary oocytes increased in women until 35 years. It decreased in groups of women aged 36–40 and 41–46 years old. The diameter and the area of primary oocytes nuclei increased in women aged 15–30 years old; later, it began to decrease. The length of follicular cells varied from 8.56 ± 0.43 μm to 8.72 ± 0.27 μm (*p* > 0.05). The height of follicular cells varied from 2.59 ± 0.27 μm to 2.7 ± 0.21 μm (*p* > 0.05).

The diameter, the area and the basement membrane thickness of primordial ovarian follicles and the diameter and the area of primary oocytes and their nuclei differed insignificantly in left and right ovaries in all age groups of women (*p* > 0.5). The length and height of follicular cells were similar in left and right ovaries of the same age group (*p* > 0.5).

**Conclusions:**

The age decreasing of morphometrical parameters begins in primordial ovarian follicles and their primary oocytes in Lithuanian women older than 35 years. The thickness of the follicular basement membrane increased with increasing age of women. No significant differences were found in the morphometrical parameters in primordial follicles of left and right ovaries in the same age group of women.

**Electronic supplementary material:**

The online version of this article (10.1186/s12958-018-0384-4) contains supplementary material, which is available to authorized users.

## Background

When ovarian follicles are formed, they enter the primordial (“resting”) stage, which persists for a period of time that varies from follicle to follicle. They can develop to primary follicles or become atretic [1]. The mechanisms responsible for the initiation of follicular growth (primordial follicle activation) or atresia and the mechanisms that permit variable timing of growth initiation are completely unknown. The non-growing primordial follicles are a resource that could be utilized or manipulated to alleviate infertility, produce contraception or delay menopause [2].

Reproductive methods, such as IVF, ICSI and embryo transfer, have a limited impact on a lack of fertilizable oocytes for women. Superovulation can increase the number of oocytes ovulated by an individual, but the response is variable and large numbers are not generally obtained. In-vitro maturation of immature oocytes from antral follicles and earlier follicle stages would increase the number of fertilizable oocytes [3].

Frozen (cryopreserved) ovarian biopsies would also provide an important source of self- and donated oocytes for the many women who do not respond well to superovulation, women with primary ovarian insufficiency, damaged ovaries due to inflammation or endometriosis, and women with premature menopause, such as young cancer patients who require fertility treatment after chemotherapy and radiotherapy [3–7].

Cryopreservation of human ovarian tissue containing immature follicles and retransplantation of cryopreserved tissue after cancer treatment can be performed successfully and healthy babies can be delivered [8–12].

Primordial follicles constitute the largest part of the ovarian follicular population. The number of primordial follicles in an ovary decreases with age [13].

A lot of scientists focus their attention on the structure and ultrastructure and changes of the ultrastructure in primordial, primary, secondary (preantral) and antral (Graafian) follicles and their oocytes in women [1, 14–18].

However, we missed detailed measurements of the size of follicles, oocytes and follicular cells and their age-related changes in all stages of human ovarian follicles.

The aim of this study was to measure primordial ovarian follicles and to determine how morphometrical parameters varied in relation with women’s age.

## Methods

### Study design

**Fig. 1:**
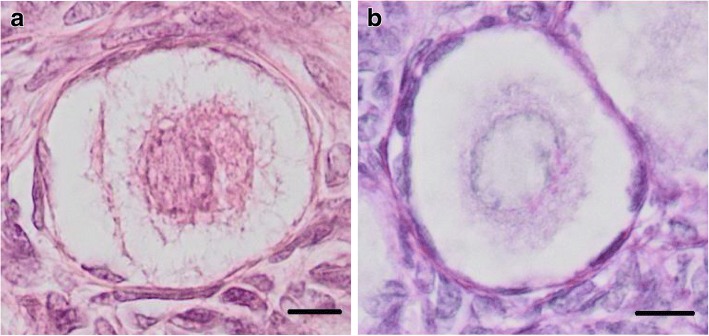
Primordial ovarian follicles in a 21 year old woman. **a** – stained with haematoxylin-eosin. **b** - stained with periodic acid–Schiff (PAS). Bar = 10 μm

The pairs of ovaries from 30 women aged 15–46 years were obtained from Kaunas Division of State Forensic Medicine Service after autopsy at least 24 h post mortem. Left ovaries were marked using the cotton thread. The material was placed into a 10% formaldehyde solution for 24 h. Women were subdivided into six age groups: 15–20 years old (*n* = 5; 15, 16, 16, 19 and 20 years old), 21–25 years old (n = 5; 21, 21, 23, 24 and 25 years old), 26–30 years old (n = 5; 26, 27, 28, 29 and 30 years old), 31–35 years old (n = 5; 31, 32, 32, 34 and 35 years old), 36–40 years old (n = 5; 36, 36, 38, 39 and 40 years old) and 41–46 years old (n = 5; 41, 43, 43, 43 and 46 years old). Only ovaries without morphological pathologies, dominant follicle, corpus luteum and follicular cysts were selected as suitable for this investigation. Each ovary was cut into 3 pieces. Only the middle piece was used for the preparation of histological slides, dehydrated in the graded ethanol series and embedded to paraffin blocks. In order to avoid the re-measurement of the same follicle, sections (4 μm of thickness) were obtained at 100 μm intervals. Histological slides were prepared according to standard methods and stained with haematoxylin-eosin (Fig. 1a). In brief, deparaffinised and rehydrated sections were immersed in Mayer’s haematoxylin solution for 7 min, and then washed in running tap water for 1 min, ammonia water for 30 s and tap water for 1 min again. The sections were stained in a 2% eosin solution for 1 min and 30 s, rinsed in tap water for 1 min, dehydrated and covered with cover glass (according to [19, 20] with our small modifications).

The periodic acid–Schiff (PAS) staining method was used for the emphasis of the follicular basement membrane (Fig. 1b) [21]. Briefly, after deparaffinisation and rehydration, the sections were immersed in a 0.5% periodic acid solution for 5 min. Then they were rinsed in distilled water and dried in a thermostat at 60 °C for 5 min. They were steeped in Schiff’s reagent (Merck) for 20 min and then rinsed in running tap water. Then the sections were stained with Mayer’s haematoxylin for 5 min, rinsed in distilled water for 5 min, and dehydrated and covered with cover glass ([19, 20] with our small modifications).

Microphotographs of primordial ovarian follicles were made from histological slides using microscope Olympus BX40 (camera Olympus XC30). Only follicles surrounded by flattened follicular cells, cut through the middle and presenting approximately equal size, rounded shape and clearly visible oocytes nuclei were selected for morphometrical analysis. Ten primordial ovarian follicles were selected from each ovary for measurement (in total, 20 follicles from each woman). Morphometrical analysis was done manually using an image analysis programme (UTHSCSA Image Tool for Windows Version 3.0) by the same person. The diameter, the area and the basement membrane thickness of primordial ovarian follicles, the diameter and the area of primary oocytes, the diameter and the area of their nuclei and the length and the height of flattened follicular cells were measured and compared in women of six age groups. Because the shape of primordial ovarian follicles, primary oocytes and their nuclei is not perfectly round, these structures were measured in three places: the widest, the narrowest and between them, and the average was calculated. The length and the height of follicular cells were measured in three cells and the average of these parameters was calculated. The basement membrane thickness was measured at five places of the same follicle and the average was calculated too. All obtained data were tabled in the Microsoft Excel 2003 programme. Using this programme, the ratios of oocyte to follicle area, nucleus to oocyte area and follicular cell height to length were calculated.

### Statistical analysis

The Statistica programme (Statistica Version 5, StatSoft inc.) Basic statistics was used for the calculation of the mean and the standard deviation. One-way ANOVA Tukey HSD post-hoc was used for statistical comparison of age groups (*p* values). The data were expressed as mean ± standard deviation (SD), and *p* < 0.05 was taken as significant.

## Results

**Fig. 2:**
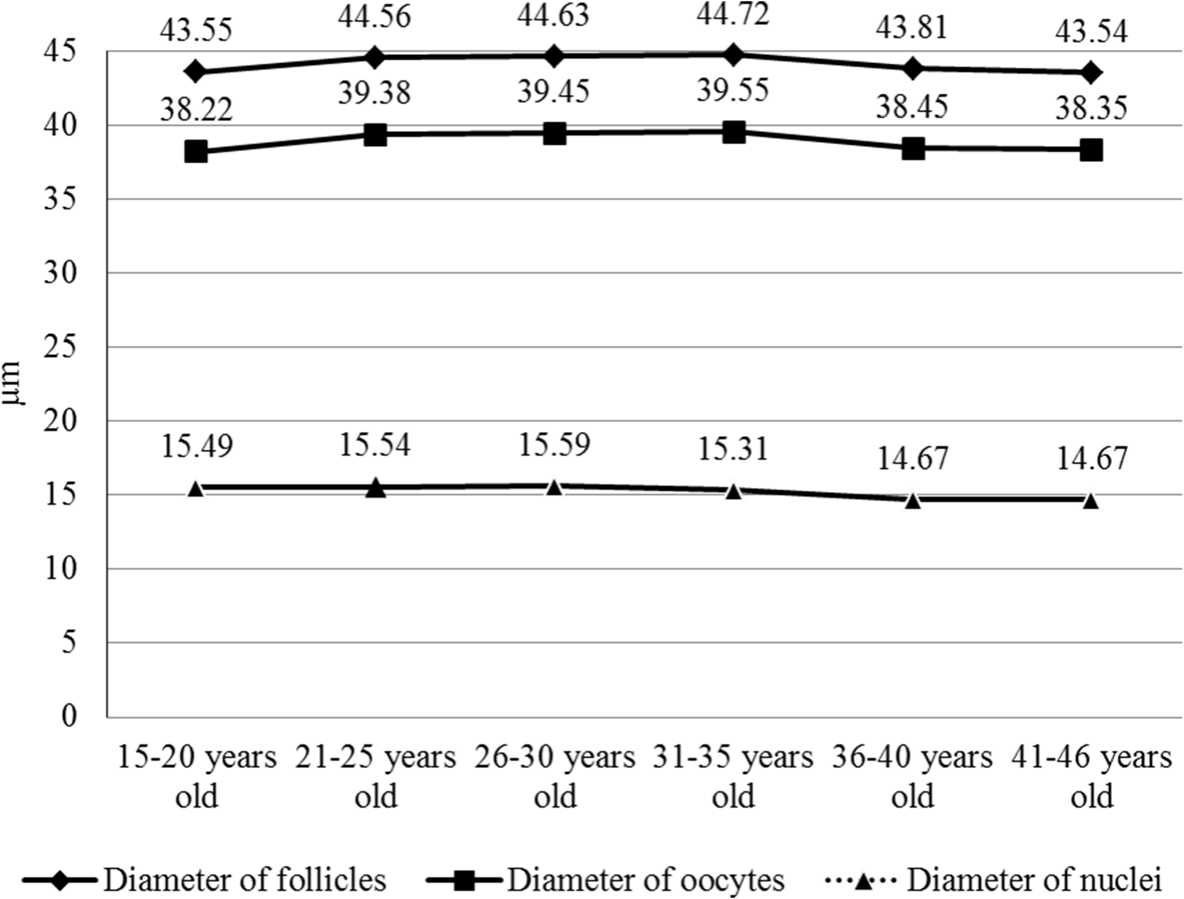
The diameter of primordial ovarian follicles, primary oocytes and their nuclei

The study of 15–46–year-old women’s primordial ovarian follicles showed that the age had a significant influence on their diameter (Fig. 2, Additional file 1: Table S1). It increased in women groups of 15–20, 21–25, 26–30 and 31–35 years old (43.55 ± 0.44 μm, 44.56 ± 0.34 μm, 44.63 ± 0.36 μm and 44.72 ± 0.38 μm, respectively). The diameter began to decrease in women of 36–40 and 41–46 years old (43.81 ± 0.56 μm and 43.54 ± 0.46 μm).

**Fig. 3:**
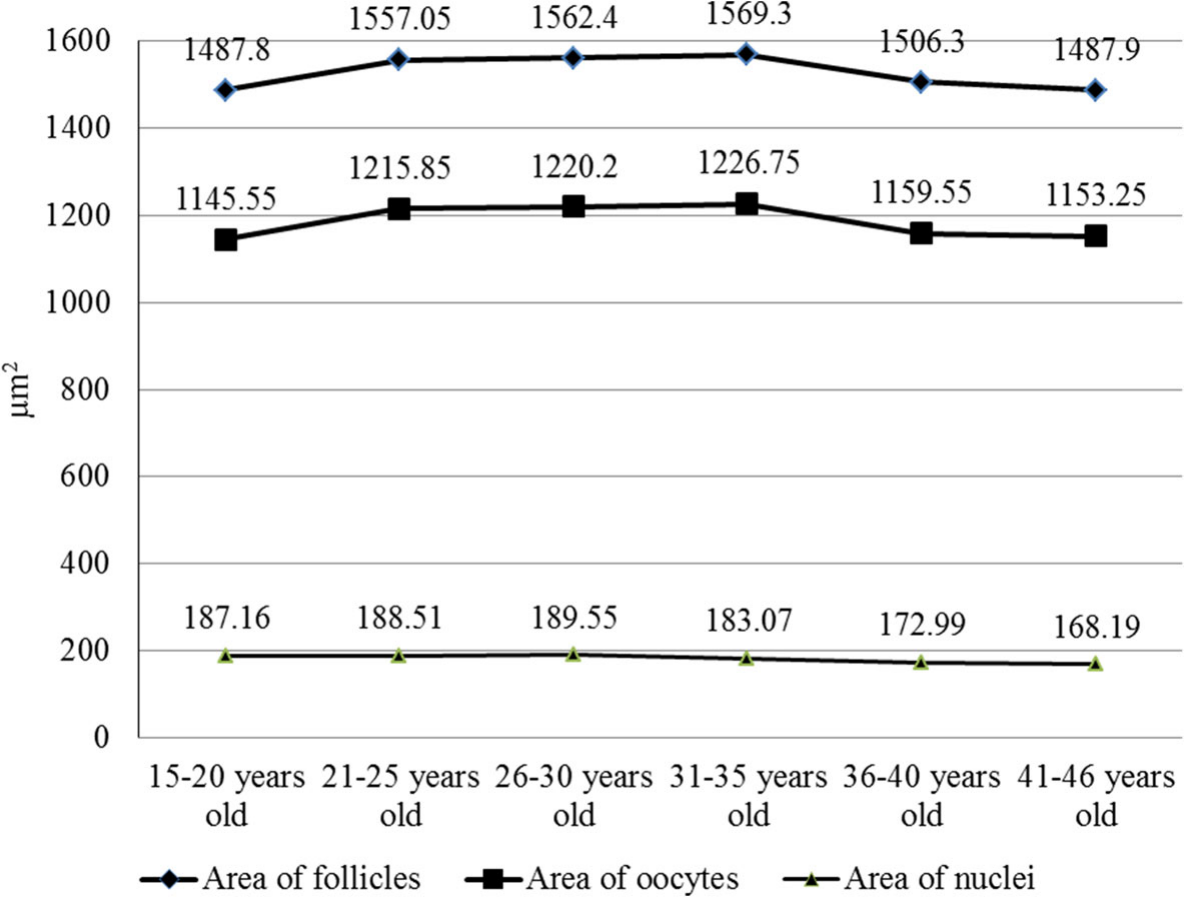
The area of primordial ovarian follicles, primary oocytes and their nuclei

Women’s age had a significant influence on the area of primordial ovarian follicles, too. It increased in women younger than 35 years old (from 1487.8 ± 32.14 μm^2^ to 1569.2 ± 28.32 μm^2^). Also, the area decreased in women groups of 36–40 and 41–46 years old (1506.3 ± 41.3 μm^2^ and 1487.9 ± 36.84 μm^2^, respectively) (Fig. 3, Additional file 1: Table S1).

The basement membrane of primordial follicles was broken and clearly visible only on slides stained by PAS. The follicular basement membrane thickness increased from 1.29 ± 0.11 μm to 1.43 ± 0.18 μm according to women’s age (Table 1).

**Table 1:** The morphometric parameters of primordial follicles in the ovaries of 15–46 year old women (mean ± SD)

Parameter	15–20 years old	21–25 years old	26–30 years old	31–35 years old	36–40 years old	41–46 years old	*p* values
Basement membrane thickness, μm	1.29 ± 0.11 a	1.34 ± 0.06	1.38 ± 0.07	1.39 ± 0.08 b	1.43 ± 0.15 c	1.43 ± 0.18 d	a:b, a:c, a:d; *p* < 0.05
Oocyte:follicle area ratio	0.77	0.78	0.78	0.78	0.77	0.78	*p* > 0.5
Nucleus:oocyte area ratio	0.16	0.16	0.16	0.15	0.15	0.15	*p* > 0.5
Follicular cells							
Length, μm	8.69 ± 0.13	8.7 ± 0.15	8.71 ± 0.12	8.72 ± 0.27	8.62 ± 0.26	8.56 ± 0.43	*p* > 0.05
Height, μm	2.62 ± 0.12	2.66 ± 0.09	2.68 ± 0.11	2.7 ± 0.21	2.59 ± 0.27	2.62 ± 0.39	*p* > 0.05
Height:length ratio	0.3	0.31	0.31	0.31	0.3	0.31	*p* > 0.5

The diameter of primary oocytes in primordial follicles was smallest in the group of women aged 15–20 years (38.22 ± 0.39 μm). It enlarged until 35 years (to 39.55 ± 0.45 μm) and then began to decrease (to 38.35 ± 0.41 μm) (Fig. 2, Additional file 1: Table S1).

The area of primary oocytes of primordial follicles increased in women until 35 years old (from 1145.55 ± 25.75 μm^2^ to 1226.75 ± 29.67 μm^2^), too. It decreased in women groups of 36–40 and 41–46 years old (1159.55 ± 22.91 μm^2^ and 1153.25 ± 26.71 μm^2^, respectively) (Fig. 3, Additional file 1). However, the oocyte to follicle area ratio remained almost the same (0.77–0.78; *p* > 0.5) (Table 1).

The age had influence on the diameter and the area of nuclei of primary oocytes in primordial ovarian follicles in 15–36-year old women. The diameter and the area of nuclei increased fractionally in 15–30-year old women (from 15.49 ± 0.2 μm to 15.59 ± 0.14 μm, and from 187.16 ± 4.84 μm^2^ to 189.55 ± 3.57 μm^2^, respectively). Later, it began to decrease significantly (to 14.67 ± 0.5 μm and 168.19 ± 13.23 μm^2^, respectively, Figs. 2 and 3, Additional file 1: Table S1). However, the nucleus to oocyte area ratio remained almost the same in the follicles of women of all ages (0.15–0.16; *p* > 0.5) (Table 1).

In primordial follicles, primary oocytes were surrounded by one layer of flattened follicular cells. The length of follicular cells did not differ significantly with age. It varied from 8.56 ± 0.43 μm to 8.72 ± 0.27 μm (*p* > 0.05). The height of follicular cells ranged from 2.59 ± 0.27 μm to 2.7 ± 0.21 μm (*p* > 0.05, Table 1).

Table 2 shows the comparison of morphometrical data of left and right ovaries in the group of women of the same age. The diameter, the area and the basement membrane thickness of primordial ovarian follicles differed insignificantly in left and right ovaries in all age groups of women (*p* > 0.5). Also, the diameter and the area of primary oocytes and their nuclei were similar in left and right ovaries (*p* > 0.5). The length and the height of follicular cells differed insignificantly in left and right ovaries, too (*p* > 0.5). The oocyte to follicle area, the nucleus to oocyte area and the follicular cell height to length ratios were the same in left and right ovaries in the same age group (0.77–0.78, 0.15–0.16 and 0.3–0.31, respectively; *p* > 0.5).

**Table 2:** The morphometric parameters of primordial follicles of left and right ovaries in 15–46 year old women (mean ± SD)

Age	Ovary	Follicles			Oocytes			Oocytes nuclei			Follicular cells			p values between the same parameter of left and right ovaries
		Diameter, μm	Area, μm^2^	Basement membrane thickness, μm	Diameter, μm	Area, μm^2^	Oocyte:follicle area ratio	Diameter, μm	Area, μm^2^	Nucleus:oocyte area ratio	Length, μm	Height, μm	Height:lengh ratio	
15–20 years	Left	43.54 ± 0.54	1486.6 ± 38.45	1.28 ± 0.12	15.5 ± 0.21	187.27 ± 5.02	0.77	15.5 ± 0.21	187.27 ± 5.02	0.16	8.62 ± 0.13	2.6 ± 0.11	0.3	*p* > 0.5
	Right	43.57 ± 0.35	1489 ± 26.45	1.3 ± 0.11	15.49 ± 0.2	187.05 ± 4.93	0.77	15.49 ± 0.2	187.05 ± 4.93	0.16	8.67 ± 0.14	2.63 ± 0.14	0.3	
21–25 years	Left	44.59 ± 0.34	1554.7 ± 24.16	1.34 ± 0.07	15.54 ± 0.15	188.48 ± 3.67	0.78	15.54 ± 0.15	188.48 ± 3.67	0.16	8.67 ± 0.17	2.65 ± 0.07	0.31	*p* > 0.5
	Right	44.52 ± 0.35	1559.4 ± 24.06	1.33 ± 0.04	15.54 ± 0.16	188.55 ± 3.85	0.78	15.54 ± 0.16	188.55 ± 3.85	0.16	8.73 ± 0.12	2.67 ± 0.11	0.31	
26–30 years	Left	44.61 ± 0.37	1560.5 ± 25.51	1.37 ± 0.07	15.59 ± 0.15	189.54 ± 3.77	0.78	15.59 ± 0.15	189.54 ± 3.77	0.16	8.71 ± 0.14	2.69 ± 0.08	0.31	*p* > 0.5
	Right	44.66 ± 0.36	1564.3 ± 25.36	1.38 ± 0.08	15.59 ± 0.14	189.56 ± 3.56	0.78	15.59 ± 0.14	189.56 ± 3.56	0.16	8.72 ± 0.09	2.67 ± 0.13	0.31	
31–35 years	Left	44.7 ± 0.4	1569 ± 30.23	1.39 ± 0.08	15.32 ± 0.4	183.33 ± 12.2	0.78	15.32 ± 0.4	183.33 ± 12.2	0.15	8.72 ± 0.3	2.7 ± 0.24	0.31	*p* > 0.5
	Right	44.73 ± 0.37	1569.6 ± 27.91	1.4 ± 0.09	15.3 ± 0.41	182.81 ± 11.56	0.78	15.3 ± 0.41	182.81 ± 11.56	0.15	8.73 ± 0.24	2.69 ± 0.19	0.31	
36–40 years	Left	43.8 ± 0.66	1505.1 ± 48.6	1.42 ± 0.13	14.82 ± 0.37	171.78 ± 10.12	0.77	14.82 ± 0.37	171.78 ± 10.12	0.15	8.6 ± 0.25	2.6 ± 0.3	0.3	*p* > 0.5
	Right	43.83 ± 0.48	1507.5 ± 35.15	1.44 ± 0.17	14.92 ± 0.48	174.2 ± 12.96	0.77	14.92 ± 0.48	174.2 ± 12.96	0.15	8.62 ± 0.27	2.58 ± 0.26	0.3	
41–46 years	Left	43.52 ± 0.55	1485.5 ± 39.29	1.45 ± 0.18	14.65 ± 0.4	167.38 ± 10.99	0.78	14.65 ± 0.4	167.38 ± 10.99	0.15	8.54 ± 0.37	2.61 ± 0.37	0.31	*p* > 0.5
	Right	43.57 ± 0.38	1490.3 ± 36.18	1.41 ± 0.19	14.69 ± 0.6	169.0 ± 15.74	0.78	14.69 ± 0.6	169.0 ± 15.74	0.15	8.58 ± 0.49	2.62 ± 0.42	0.31	

## Discussion

In this study, we wanted to measure primordial ovarian follicles and their primary oocytes and to compare how the morphometrical parameters varied in relation to women’s age in Lithuania. Starting this research, we *confronted with* the lack of research material. Within 10 years, we received ovaries from 49 dead women (15–46 years old only). Therefore, our study was very extended. Only ovaries from 30 women were suitable for our research (without corpus luteum, dominant follicle or follicular cysts). Also, there were cases when we received only one ovary. Therefore, we could not compare the morphometrical parameters of primordial follicles of left and right ovaries in the same women. Besides, the investigation was impeded by post mortem changes in the ovaries.

We aimed at comparing morphometrical parameters of follicles in women of different countries. However, in the literature, we found scarce data on morphometrical characteristics of primordial ovarian follicles in women from various countries.

In the USA, Griffin with co-authors [22] maintained that the mean diameter of human primordial ovarian follicles and primary oocytes was 44 μm and 36 μm, respectively. In the Netherlands, the area of oocytes was 850 ± 244 μm^2^ in primordial follicles of young women’s (26–32 years old) ovaries and 927 ± 258 μm^2^ in those of advanced-age women (39–45 years old). The area of oocytes nuclei was 199 ± 97.7 μm^2^ and 197 ± 94 μm^2^, respectively. The mean nucleus to oocyte ratios were 0.23 ± 0.09 and 0.21 ± 0.08, respectively [15, 16].

In France, the diameter of human primordial ovarian follicles was 35.4 ± 6.2 μm, the diameter of oocytes was 32.1 ± 6.0 μm and the diameter of oocytes nuclei was 16.1 ± 6.1 μm [13].

We found only one source of literature which in detail compared morphometrical parameters of follicles in the ovaries of women of the reproductive age. Westergaard and co-authors [23] from Denmark proposed that the diameter of primordial ovarian follicle and its oocytes increased in women of 13–27 years old (from 39.0 ± 0.4 μm to 41.9 ± 03 μm and from 34.3 ± 0.4 μm to 37.0 ± 0.3 μm, respectively); then it began to decline (to 39.4 ± 0.3 μm and 35.1 ± 0.4 μm, respectively). The maximal diameter of oocytes nuclei was found in 13–20-year old women’s oocytes (18.9 ± 0.2 μm). The oocytes of < 13-year old girls had the smallest nuclei (17.8 ± 0.2 μm).

The results of our study correspond partially to Westergard et al.’s [23] results. The diameter and the area of primordial ovarian follicles, the diameter and the area of primary oocytes and their nuclei increased in Lithuanian women from 15 to 35 years, and then these parameters declined with age.

According to our study, the hypothesis can be made that the negative changes of aging begin in primordial ovarian follicles and their primary oocytes in Lithuanian women older than 35 years. Therefore, it can be recommended that women should plan pregnancy up to 35 years of age. Subsequently, negative changes begin in oocytes, and this may be one of the causes of decreased fertility and increased birth defects.

In results of our study, the morphometrical parameters of Lithuanian women’s primordial ovarian follicles were similar to those of women in the USA, but different than those of women in France and Denmark. Primary oocytes in Lithuanian women’s primordial ovarian follicles had a larger diameter and area in comparison with these parameters in women from the USA, France and Denmark. Primary oocytes in Lithuanian women had smaller nuclei than in French, Danish and Dutch women.

It can be concluded that morphometrical parameters of primordial follicles and their primary oocytes can vary according to the country and the region in which women live.

## Conclusions

1. The diameter and the area of primordial ovarian follicles, the diameter and the area of primary oocytes and their nuclei increased in Lithuanian women from 15 to 35 years; then these parameters began to decrease.
2. The thickness of the follicular basement membrane increased in primordial ovarian follicles of 15–46-year old Lithuanian women with age.
3. The length and the height of follicular cells differed insignificantly in different age groups of Lithuanian women (*p* > 0.05).
4. No differences were observed in the morphometrical parameters of primordial follicles, primary oocytes and their nuclei and follicular cells of left and right ovaries in women of all age groups (*p* > 0.5).

## Additional file

Additional file 1: **Table S1.** Morphometrical parameters of primordial ovarian follicles in 15–46 year old women described in the Figs. 2 and 3 (mean ± SD). (XLS 34 kb)
